# Optical Coherence Tomography Characteristics for Differentiating Scars in Type 1 (Polypoidal Choroidal Vasculopathy (PCV)) and Type 2 (Classical) Macular Neovascularization (MNV) in Age-Related Macular Degeneration (AMD)

**DOI:** 10.7759/cureus.62593

**Published:** 2024-06-18

**Authors:** Brughanya Subramanian, Meenakshi Kumar, Parveen Sen, Rajiv Raman

**Affiliations:** 1 Department of Ophthalmology, Shri Bhagwan Mahavir Vitreoretinal Services, Sankara Nethralaya, Chennai, IND

**Keywords:** retina, oct, scar, polypoidal choroidal vasculopathy, choroidal neovascular membrane

## Abstract

Purpose: This study aimed to assess the optical coherence tomography (OCT) characteristics for differentiating scars in the scarred stages of macular neovascularization (MNV) in age-related macular degeneration (AMD).

Methods: Medical records of 20 patients, 10 in each group with type 1 and type 2 MNV, were selected for the study. Participants chosen were above 50 years of age and underwent comprehensive eye examination alongside indocyanine green angiography (ICGA), fundus fluorescence angiography (FFA), and Spectralis optical coherence tomography (SOCT) (Heidelberg Engineering, Germany), respectively. The qualitative and quantitative OCT measurements, such as the frequency of outer retinal tubulations, presence of cystoid spaces, scar area, choroid thickness, retinal thickness, presence of disorganization in retinal layers (DRIL), foveal contour, and involvement of retinal layers in the scar, were meticulously evaluated and compared between the two groups.

Results: Significant disparities between type 1 MNV and type 2 MNV in choroidal thickness were identified in the nasal and superior quadrants within 1 mm, in the superior quadrant within 3 mm, and in all quadrants except the inferior quadrant within 6 mm. Overall, type 2 MNV showed thinner choroid than type 1 MNV.

Conclusion: Although there are several overlapping features noticed between the groups, the OCT was able to pick up characteristic features that aid in differentiating type 1 (polypoidal choroidal vasculopathy (PCV)) and type 2 (classic) MNV in AMD. This precise differentiation has the potential to assist ophthalmologists in making well-informed decisions, thereby enhancing patient care.

## Introduction

Age-related macular degeneration (AMD) is a condition typically affecting individuals over 50 years old, characterized by the deterioration of the macula's structure and function. A key feature of AMD is the accumulation of extracellular deposits, such as subretinal drusenoid deposits, basal linear deposits, and basal laminar deposits. Additionally, these eyes may exhibit neovascularization or atrophy [1]. Polypoidal choroidal vasculopathy (PCV) is a variant of type 1 macular neovascularization (MNV) frequently observed in individuals of Asian descent. Indocyanine green angiography (ICGA) reveals a branching vascular network with varying numbers of aneurysmal dilations at the outer edge of the expanding lesion. The internal structure of these aneurysmal formations, commonly referred to as polyps, remains a topic of debate [1]. However, it shows better outcomes with Eylea or newer-generation anti-vascular endothelial growth factor (anti-VEGF) treatments, possibly in combination with photodynamic therapy (PDT). It is also important to note that scarred AMD often does not require treatment, as the structural changes do not respond well to therapies and the condition progresses slowly with a low risk of hemorrhage if left untreated [2]. The EVEREST study report no. 2 has outlined the criteria to identify type 1 MNV based on the identification of polypoidal lesion and branching vascular network as seen on ICGA and optical coherence tomography (OCT) [3].

Advanced AMD is characterized by the presence of type 1 MNV which eventually becomes a disciform scar [4]. Likewise, type 1 MNV in the late stage is characterized by scarring of the lesion [5]. At this stage of scarring, the features exhibited by both diseases appear similar. However, it is essential to distinguish the two as this has implications regarding the risk of fellow eye involvement and in the decision of pacing the follow-up for the patient. Previous studies have been performed to distinguish type 1 MNV and type 2 MNV based on OCT features [6]; however, this included participants presenting in the active stage of the disease. During the active stage, polypoidal lesion may not be visible on the ICGA which makes the diagnosis challenging. To the best of our knowledge, this is the first study to assess the OCT features of both type 1 and type 2 MNV in their scarred states. Given that scar and atrophy are the end results of AMD, it is crucial to understand their distinct characteristics. In type 1 MNV, there is a higher risk of recurrence, bleeding, and formation of new polyps; therefore, type 1 MNV requires more frequent evaluation. Although the treatment for both conditions involves anti-VEGF therapy, different investigations are important to accurately identify the lesions. The purpose of this study is to evaluate the OCT-based differences between scarred type 1 and type 2 MNV of AMD to facilitate treatment of the fellow eye.

## Materials and methods

This study involves a retrospective review of electronic medical records of patients with type 1 and type 2 MNV in AMD between January 2016 and December 2016 to identify "scarred type 1 MNV" and "scarred type 2 MNV." The study was approved by the Vision Research Foundation Institutional Review Board, and the approval number is 632-2017-P. All the research adhered to the tenets of the Helsinki Declaration. Written informed consent was obtained from the enrolled subjects, and all authors had access to subject information for data collection. The research did not receive any funding, and there are no conflicts of interest reported by any of the authors.

The selection was restricted to patients aged 50 years and above, who had undergone a comprehensive eye examination alongside dilated fundus examination, fundus fluorescence angiography (FFA), ICGA, and Spectralis optical coherence tomography (SOCT) (Heidelberg Engineering, Germany).

For group 1 recruitment, individuals exhibiting type 2 MNV due to AMD based on FFA and clinical examination findings were included. Specifically, those with a neovascular complex located in the subretinal space, above the level of the retinal pigment epithelium (RPE), may be associated with subretinal hyperreflective material and separation of the neurosensory retina from the RPE. OCT angiography showed vascular elements above the RPE [1].

Similarly, group 2 comprised patients exhibiting characteristics of type 1 MNV based on FFA and ICGA features, and the patients were chosen according to the following criteria: neovascular complexes originating from the choroid, which are imaged with OCT as an elevation of the RPE containing material with heterogeneous reflectivity, with potential visible vascular elements and nodules; OCT angiography displaying vessels below the level of the RPE, where dilated vascular elements are visible at the outer edge of the lesion; FFA showing stippled hyperfluorescence over the elevated RPE area, which expands and coalesces in the later stages; and ICGA revealing a branching vascular network with aneurysmal dilations [1].

The analysis encompassed a total of 20 eyes, with an equal distribution of 10 eyes each from subjects diagnosed with scarred type 1 and type 2 MNV. The definition of scarred type 1 and type 2 MNV involved the absence of indications such as subretinal fluid, subretinal hemorrhage, and serous pigment epithelial detachment (PED) as observed on OCT or during clinical examination, combined with a lack of reported vision deterioration over a span of three to six months. Patients exhibiting conditions like geographic atrophy or those with a history of trauma or scarring from other causes were excluded from the study. The analysis encompassed both individuals who were yet to receive treatment and those who had undergone treatment and eventually progressed to the scarring stage.

SOCT imaging

**Figure 1:**
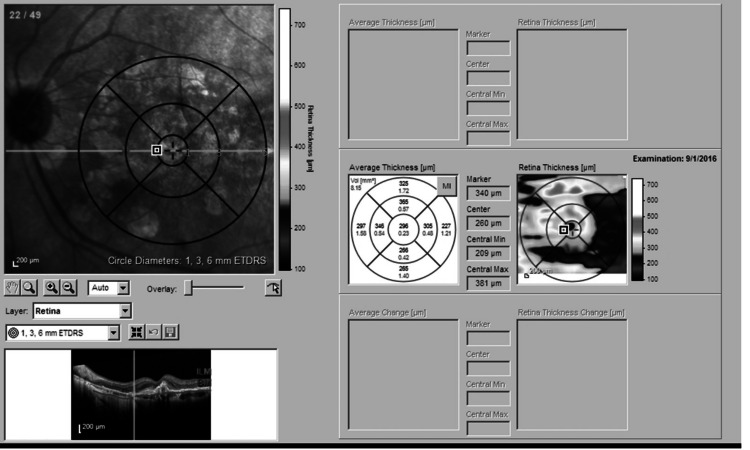
The ETDRS grid used as a landmark for 1, 3, and 6 mm in all four quadrants ETDRS: Early Treatment Diabetic Retinopathy Study

Only OCT images assessed with SOCT+Heidelberg retinal angiogram (HRA) were included in this study. All patients underwent SOCT (Heidelberg, Germany) OCT scanning pattern 512×97 A-scans covering 20×20° centrally. Enhanced depth imaging (EDI) scan was used to analyze the images and take measurements. The Early Treatment Diabetic Retinopathy Study (ETDRS) circle was superimposed over the centered macula, and the 1, 3, and 6 mm circle landmarks were used to measure thickness across the four quadrants as shown in Figure 1. The images were marked by a grader and verified by a senior retina specialist. The decisions of both specialists were mutual for grading the disease and inactivity. Disagreements between the specialists were settled down with discussion.

Quantitative SOCT factors

**Figure 2:**
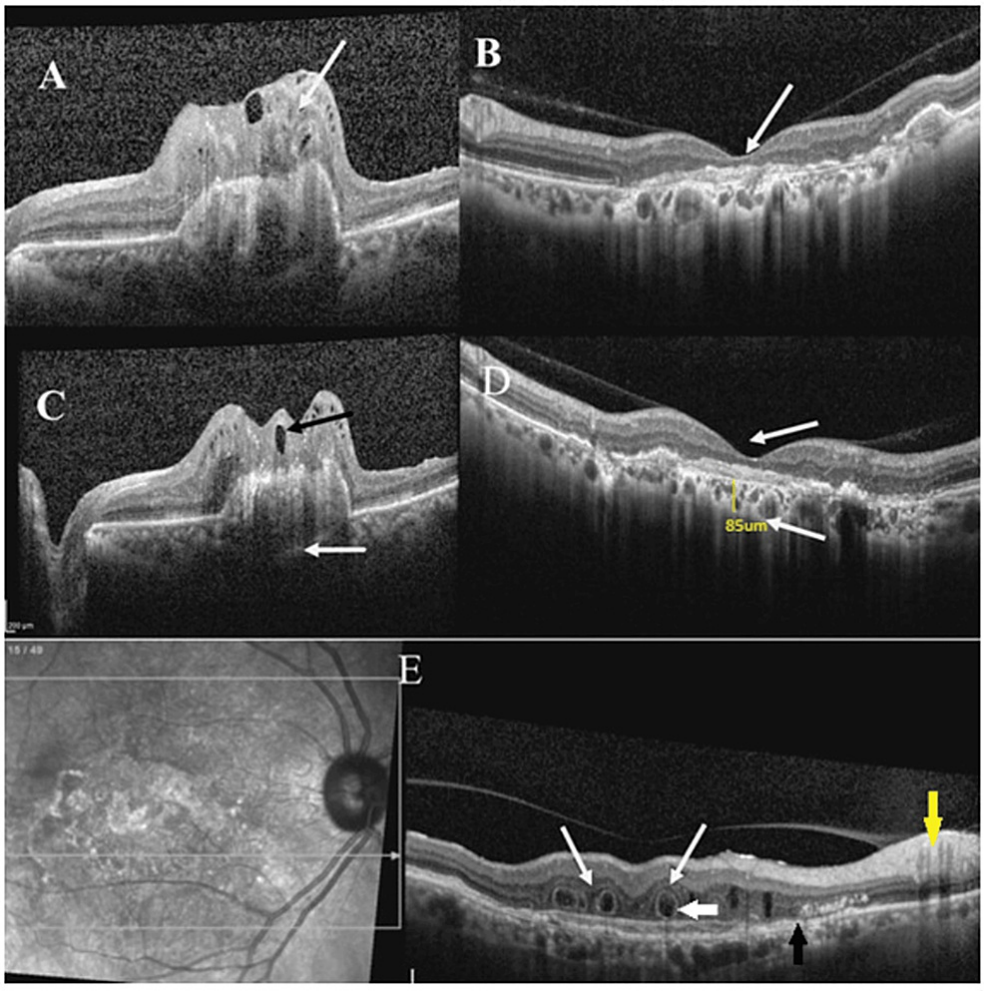
OCT images in AMD: (A) an arrow pointing towards the disorganized inner layers; (B) intact retinal layers, absence of disorganization of inner retinal layers; (C) the arrow above the RPE pointing towards the distorted foveal contour and the arrow below the RPE pointing towards the thickened choroid; (D) the arrow above the RPE pointing towards the maintained foveal contour and the arrow below the RPE pointing towards the thin choroid; and (E) the white arrow pointing towards the outer retinal tubulations with hyperreflective surrounding, the black arrow pointing towards the RPE alterations, and the yellow arrow pointing towards the disorganization of inner retinal layers AMD: age-related macular degeneration; RPE: retinal pigment epithelium

Quantitative factors such as the number of outer retinal tubulations (ORTs), the number of cystoid spaces, the area of the scar, the choroidal thickness, the inner retinal thickness, and the outer retinal layer thickness were assessed. ORTs are hyporeflective tube-like spaces seen in the outer retinal layer, with hyperreflective surrounding as seen in the OCT [7] as shown in Figure 2. 

**Figure 3:**
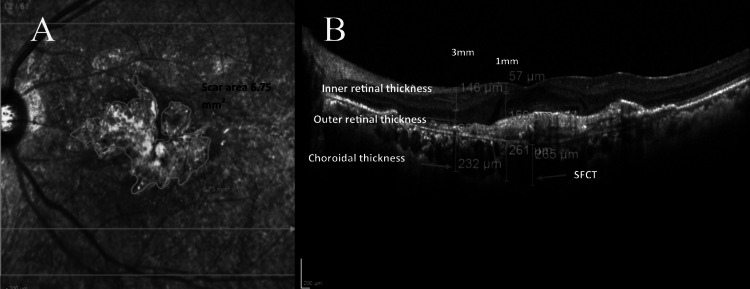
The quantitative measurements: (A) showing the area of scar measurement and (B) showing the inner and outer retinal thickness, the choroidal thickness measured at 1 and 3 mm junction of the EDTRS grid, and the SFCT measured ETDRS: Early Treatment Diabetic Retinopathy Study; SFCT: subfoveal choroidal thickness

The cystoid space area was assessed by manually marking the borders of the cystoid space in the SOCT. The area of the scar was assessed by marking the border of the scar in the fundus image shown on the OCT screen. The inner retina layers were defined as the layers between the internal limiting membranes and the inner nuclear layer. The outer retinal layers were defined as the layers between the outer plexiform layers and the photoreceptor layer as shown in Figure 3.

Qualitative SOCT factors

The qualitative analysis included the assessment of disorganization in retinal layers (DRIL), layers involved in the scar, and foveal contour. DRIL was said to be present when the boundaries between two layers of the inner retina were ill-defined or indistinguishable [8]. Intact foveal contour is considered to be maintained when the foveal dip is present [9]. Layers of the retina involved in the scar were identified by the layer seen clearly above the scar at the fovea.

Statistical analysis

The data was analyzed using IBM SPSS Statistics for Windows, Version 24.0 (Released 2016; IBM Corp., Armonk, New York, United States). The normality assumption was checked with the Shapiro-Wilk test. Qualitative data was analyzed using Pearson's chi-squared test. Parametric data was analyzed using an independent t-test, while non-parametric data was analyzed using the Mann-Whitney test.

## Results

Qualitative factors

The qualitative factors assessed are listed in Table 1. In the type 2 MNV group, 50% of the cases with scar involved the outer nuclear layer, and in the remaining 50% of the cases, the scar tissue involved the inner layers. In the type 1 MNV group, the scar tissue involved the outer nuclear layer in 40% of the cases and the inner nuclear layer in 60% of the cases. The foveal contour was maintained in 60% of type 2 MNV cases, while in type 1 MNV, the foveal contour was maintained in 90% of cases. These changes were not statistically significant across the groups. However, the disorganization of the inner retinal layers was found to be present in 60% of type 2 MNV and in type 1 MNV in only 10% of cases. This difference was found to be statistically significant (p=0.019).

**Table 1:** Qualitative assessment of the OCT-based features A p-value <0.05 is considered significant *MNV: macular neovascularization; †PCV: polypoidal choroidal vasculopathy; ‡ONL: outer nuclear layer; **INL: inner nuclear layer; OCT: optical coherence tomography

Factor assessed			P-value
Disorganization in the retinal layers			
	Yes	No	0.019
Type 2 MNV (classic)^*^	6	4	
Type 1 MNV (PCV)^†^	1	9	
Total	7	13	
Layers involved in the scar			
	ONL^‡^	INL^**^	0.653
Type 2 MNV (classic)^*^	5	5	
Type 1 MNV (PCV)^†^	4	6	
Total	9	11	
Foveal contour			
	Maintained	Not maintained	0.121
Type 2 MNV (classic)^*^	6	4	
Type 1 MNV (PCV)^†^	9	1	
Total	14	5	

Quantitative factors

The frequency of ORTs, the number of cystoid space and its area, and the area of the scar in type 1 and type 2 MNV groups were compared as shown in Table 2.

**Table 2:** Comparison of OCT-based features between the groups A p-value <0.05 is considered significant MNV: macular neovascularization; PCV: polypoidal choroidal vasculopathy; ORT: outer retinal tubulation; Std. dev.: standard deviation; SFCT: subfoveal choroidal thickness; OCT: optical coherence tomography

	Type 2 MNV (classic)			Type 1 MNV (PCV)			
Factors	Mean	Median	Std. dev.	Mean	Median	Std. dev.	P-value
ORT	3.78	1	5.84	11.33	5	14.612	0.258
Cystoid spaces	5.22	3	8.288	3.56	0	4.419	1
Area of cyst (mm^3^)	0.044	0.01	0.0596	0.0422	0	0.101	0.548
Area of the scar (mm^3^)	2.946	3.12	1.834	4.932	4.28	3.742	0.156
SFCT	166.6	165	52.623	233.7	230	90.348	0.07

The number of ORTs was higher in the type 1 MNV group (by a mean of 37.5%); however, this was not statistically significant. The cystoid spaces were marginally higher in the type 2 MNV group than in the type 1 MNV group (8.3%). The area of the cystoid space and the scar in the two groups were found to be similar. Although the difference in the area of the scar was found to be statistically insignificant, the area of the type 1 MNV scar was found to be larger and more widespread (9.93%). The subfoveal choroidal thickness (SFCT) was also compared between the groups and found to be higher in the type 1 MNV group by a mean thickness of 67 microns; however, it was found to be statistically insignificant.

Table 3 shows the demographic details in both groups. There was no difference between the groups, in terms of age (MD: 3.5 years), duration of symptoms (MD: 0.173 months), gender, refractive error range, and best corrected visual acuity (p>0.05).

**Table 3:** Demographic details of the subjects MNV: macular neovascularization; PCV: polypoidal choroidal vasculopathy; *Type 1 MNV: classic; †Type 2 MNV: PCV

	Type 2 MNV*	Type 1 MNV†	P-value
	10	10	
Age (in years)	67.60±9.14	71.10±11.98	0.472
Duration of visual symptoms (in months)	2.05±1.78	2.23±1.59	0.853
Gender (males/females)	5/5	6/4	0.653
Refractive error range (in diopters)	-2 to 4D	-1 to 4D	0.973
Visual acuity	6/24-6/171	6/9-6/181	0.442

The choroidal and the retinal thickness were compared between the type 1 MNV group and the type 2 MNV group in all four quadrants, and the mean differences are displayed in Table 4. The choroidal thickness was found to be significantly thicker in the type 1 MNV group in the superior (MD: 53.9 mm) nasal quadrants (MD: 57.8 mm) in the 1 mm ETDRS circle, superior quadrant (MD: 71.1 mm) in the 3 mm ETDRS circle, and nasal (MD: 67.3 mm), temporal (MD: 60.8 mm), and superior (MD: 68.7 mm) quadrants in the 6 mm ETDRS circle in OCT. The choroidal thickness in the inferior quadrant was found to be statistically insignificant across the two groups (mean thickness difference less than 50 mm). However, there was no statistically significant difference in the inner and outer retinal layer thickness in both groups.

**Table 4:** Comparison of retinal and choroidal thickness of type 1 and type 2 MNV in AMD The significant values are highlighted in bold MNV: macular neovascularization; AMD: age-related macular degeneration; Type 2 MNV: classic; Type 1 MNV: PCV; N: nasal; T: temporal; S: superior; I: inferior

Factor assessed	Quadrant	1 mm			3 mm			6 mm		
		Type 2 MNV	Type 1 MNV	P-value	Type 2 MNV	Type 1 MNV	P-value	Type 2 MNV	Type 1 MNV	P-value
Choroidal thickness	N	144.4±32.12	202.2±69.76	0.096	125.6±34.71	185±94.16	0.151	90.4±45.38	157.7±77.5	0.049
	T	159.8±57.41	216.6±69.51	0.053	169.4±63.84	209.4±66.17	0.121	133.9±44.19	194.7±51.32	0.012
	S	176.5±50.18	230.4±84.15	0.326	169±44.98	240.1±77.99	0.064	161.2±45.52	229.9±65.90	0.008
	I	180.3±77.62	227.7±91.30	0.273	178.7±66.25	209.4±80.90	0.496	171.1±72.44	217.4±67.14	0.082
Inner retinal thickness	N	163.4±113.86	131.8±65.06	0.734	194.4±29.07	207.6±56.72	0.650	159.3±30.11	145.1±28.69	0.257
	T	161±136.39	113±31.52	0.496	145.2±55.57	155.8±51.86	0.325	121.66±27.87	128.5±43.05	0.734
	S	182.2±12.94	152.1±68.52	0.850	179.7±37.93	174.9±28.16	0.970	146.7±42.63	132.7±22.58	0.496
	I	185.7±83.04	152.6±89.14	0.256	202.2±57.61	180.2±41.36	0.290	156.6±62.26	130±23.56	0.326
Outer retinal thickness	N	81.7±58.01	83.2±138.70	0.112	90.6±44.28	100.6±44.4	0.545	76.4±43.83	91.9±37.45	0.597
	T	82.44±22.87	102.4±146.24	0.406	89.44±34.18	98±34.16	0.273	68.88±22.4	56.9±77.21	0.364
	S	70.1±48.70	118.7±148.70	0.597	78.7±32.21	116.3±137.72	0.910	74±29.76	88±27.09	0.545
	I	66.8±32.95	71.4±74.41	0.650	72.9±52.95	83±57.52	0.879	57.2±33.27	65.3±40.44	0.520

## Discussion

In our study, the analysis of OCT features between the type 1 and type 2 MNV in AMD in the scarred stage was assessed to identify the differences in their morphology both qualitatively and quantitatively. We found two morphological features which can significantly differentiate them: increased choroidal thickness in the type 1 MNV scar and higher preponderance of disorganization of the inner retinal layers in the type 2 MNV scar.

While type 1 and type 2 MNV of AMD may exhibit numerous overlapping clinical presentation features, their underlying pathogenesis and origins are fundamentally distinct [10]. Furthermore, there exists dissimilarity in the expression of VEGF between these two conditions, leading to variations in treatment approaches and subsequent outcomes. The VEGF expression, which gives rise to disruptions in the RPE, the enlargement of blood vessels within the inner nuclear layer, and the dysgenesis of the outer nuclear layer, has previously been documented specifically within the type 2 MNV group [11]. The extent of VEGF expression could potentially influence the layer of blood vessel involvement, whether in the inner nuclear layer or the outer nuclear layer.

In this study, both groups exhibited involvement of both retinal layers, underscoring the variance in VEGF expression. The preservation of the foveal contour was more prominent in the type 1 MNV group (90%) than in type 2 MNV, attributed to the prevailing sub-RPE neovascularization in type 1 MNV [12]. When neovascularization occurs above the RPE level, it tends to involve the inner retinal layers, thereby disrupting the contour and inducing disorganization in these layers. Previous research has indicated that in type 2 MNV, disorganization ranges from the choroid to the sub-RPE space or pre-RPE, whereas in type 1 MNV, it consistently remains sub-RPE. This distinction potentially elucidates the higher occurrence of inner retinal layer disorganization in the type 2 MNV group, as opposed to the type 1 MNV group, alongside marginally reduced inner retinal thickness in type 1 MNV [12,13].

While the area of scarring was slightly greater in type 1 than type 2 MNV, despite not achieving statistical significance, from a clinical standpoint, it signifies that type 1 MNV scars extend from the macular to the extramacular region, even in cases where the lesion is primarily macular. Conversely, the type 2 MNV scar predominantly remained within the macular region within this cohort.

In this current study, retinal and choroidal thickness were compared between the two groups, and there were a few sectoral differences noted in the type 1 MNV group. The thickness was found to be higher in nasal, superior quadrants across all the 1, 3, and 6 mm ETDRS circle, and in the 6 mm circle, the temporal choroidal thickness was also found to be significant. While literature indicates notable differences in choroidal thickness between type 1 and type 2 MNV, there exists a dearth of studies that examine thickness post-reduction of activity in both groups. Moreover, SFCT has predominantly been the focus of investigation on type 1 MNV, existing within the pachychoroid spectrum of diseases, which is marked by increased choroidal thickness during its active phase, a distinguishing feature between type 1 and type 2 MNV in AMD. In this study, it was observed that even in the scarred state, choroidal thickness remained elevated within the treated group, reaffirming the persistent nature of this characteristic.

The choroidal thickness difference between the groups emphasizes the underlying choroidal vascular dilation in type 1 MNV due to the increased permeability and hyperpermeability of the choroid, whereas in type 2 MNV, there is more atrophic thinning secondary to hypoxia and resulting reduction in choroidal thickness [14]. In prior studies, variations in choroidal thickness between these two groups were noted across all four quadrants, displaying significant differences. However, our present study observed that, within both groups, the nasal quadrant exhibited the least thickness, followed by the inferior and superior quadrants and, finally, the SFCT within the type 2 MNV group. In the case of the type 1 MNV group, the temporal, superior, and inferior quadrants preceded the SFCT in terms of thickness. 

The ORTs are generally associated with outer retinal damage. Few studies have shown the increased appearance of ORTs in many neovascular disorders; however, its increased appearance is noted in AMD [15]. Another study discussed the delayed emergence of ORTs in type 1 MNV compared to type 2 MNV, with the majority being observed after a duration of 36 months [16]. In this study, the number of ORTs was higher in type 1 MNV (statistically insignificant) despite the duration of the disease being similar in both groups.

Recognizing cystoid macular degeneration as distinct from cystoid macular edema (CME) is crucial, especially since CME can appear in some exudative AMD eyes undergoing intravitreal ranibizumab treatment and may not indicate type 2 MNV activity. The cystoid spaces in cystoid macular degeneration are intraretinal and represent exudative changes within the retina, usually accompanied by retinal thickening when active. These spaces may persist even after the exudation subsides. Unlike exudative cysts, which typically have a biconvex shape, degenerative pseudocysts are uniquely square-shaped. These pseudocysts may signify an intermediate stage of retinal atrophy, marked by Müller cell loss, potentially developing before the neurosensory retina begins to thin [17].

In this study, the number of cystoid spaces was seen equally in both groups even in the scarred state representing the irrevocable damage to the retina [18]. The area occupied by the cystoid space was also found to be similar between the groups. The area of the scar was found to be marginally higher in type 1 MNV than type 2 despite not being statistically significant; clinically, it indicates the location and the origin of the type 1 MNV scar which extends from the macular to the extramacular region even when the lesion is predominantly macular, whereas the type 2 MNV scar in this cohort was found to be predominantly macular.

Although the choroidal thickness showed a topographical difference between the groups, the SFCT was found to be similar in the two groups. This could be because most of the subjects (60%) in this group were treated with injections and previous studies have reported the reduction of choroidal thickness with their use specific to SFCT suggesting recovery and reduction in stromal edema of the choroid [19]. McDonnell et al. [20] reported that the choroidal thickness reduced with time in the type 2 MNV group; however, anti-VEGF injections had no effect on them. This could be the reason why the SFCT despite being higher didn't show significance in between the groups and quadrant-wise choroidal thickness.

Study limitations

Our study has some limitations. Firstly, the study included only 20 participants. This was a pilot study to observe the changes between the groups; further studies are required to corroborate these findings to have clinical implications. Secondly, given the retrospective nature of the study, we could not account for many factors such as the time of measurements and refractive error matching between the groups. Lastly, one of the possible measurement biases is that we have not centered the EDTRS grid manually, and this misalignment may result in circles that are not corresponding and comparable. Since we only used the OCT from Spectralis, we may have lost some participants who were seen in the follow-up visits on the other OCT devices used at the hospital. Future directions for this study would include a bigger sample size to see if these findings could be replicated.

## Conclusions

Distinct morphological variations are evident in the scarred stages of type 1 and type 2 MNV, as detected through OCT. A qualitative OCT assessment revealed a higher presence of DRIL in type 2 MNV compared to type 1 MNV. While the number of layers involved in the scar was greater in type 2 MNV than in type 1, this difference was not statistically significant. Similarly, a poor foveal contour was more frequently observed in type 2 MNV than in type 1 MNV, but this difference was also not significant. Overall, SFCT was significantly thinner in the type 1 MNV group compared to the type 2 MNV group. However, zone-wise analysis showed a significantly thicker choroid in the type 1 MNV group in the nasal, temporal, and superior quadrants. Inner and outer retinal thickness were predominantly higher in the type 1 MNV group than in type 2, although these results were not statistically significant. Our findings suggest that OCT is useful for assessing characteristics in the scarred stage of type 1 and type 2 MNV in AMD. Nonetheless, it should be noted that a substantial portion of the morphological characteristics might intersect between the two conditions.
